# Supplementary material for: Difference in miRNA Expression in Functioning and Silent Corticotroph Pituitary Adenomas Indicates the Role of miRNA in the Regulation of Corticosteroid Receptors
Source: Int J Mol Sci. 2022 Mar 5;23(5):2867. doi: 10.3390/ijms23052867 (PMC8911444; doi:10.3390/ijms23052867)
Supplement: Supplementary file 1 [file ijms-23-02867-s001.zip › ijms-1596079-supplementary.pdf]

Table S1: Detail patients' characteristics

Patient	gender	age	USP8 mutation (0/1)	USP48 mutation (0/1)	Functionlal status	cortisol 08.00 (ug/dl) - 6,2-19,4 ug/dl	ACTH 08.00 (pg/dl) (10-60 pg/ml)	UFC (4,3-176 ug/24h)	cortisol 24.00 - single sleeping midnight serum cortisol level (<1,8ug/dl)	late night salivary cortisol (ug/dl, <0,21 ug/dl)	Preoperative treatment	Knosp grade	micro vs. MAKROadenoma	tumor size (mm)	ACTH (IHC)	GH (IHC)	TSH (IHC)	FSH (IHC)	LH (IHC)	α-subunit (IHC)	Ki-67 (IHC)	Patomorfological diagnosis	tumor tissue content in the sample (%)
C1	f	50	0	0	Cushig's disease	29.1	39.4	439	19.8	na	none	4	M	21x16x20	1	0	0	0	0	0	<1%	densly granulated corticotroph adenoma	97
C2	f	66	0	0	Cushig's disease	25.64	61	713	21.2	na	none	4	M	26x14x15	1	0	0	0	0	0	>5%	sparsely granulated corticotroph adenoma	98
C3	m	42	0	0	Cushig's disease	23.6	82.3	626.58	21.4	na	unknown	2	M	15x12x14	1	0	0	0	0	0	<1%	densly granulated corticotroph adenoma	97
C4	f	37	0	0	Cushig's disease	16.6	36.9	639	15.9	na	none	2	m	7x10x10	1	0	0	0	0	0	<3%	densly granulated corticotroph adenoma	99
C5	f	24	0	0	Cushig's disease	17.46	65.9	520	12.9	na	none	1	M	20x20x20	1	0	0	0	0	0	<3%	sparsely granulated corticotroph adenoma	98
C6	f	40	0	0	Cushig's disease	26.4	90	497	19.4	na	none	1	M	26x18x20	1	0	0	0	0	0	<1%	densly granulated corticotroph adenoma	96
C7	f	61	0	0	Cushig's disease	38.6	112	739	29.1	na	none	4	M	25x32x15	1	0	0	0	0	1	<3%	sparsely granulated corticotroph adenoma	98
C8	m	31	1	0	Cushig's disease	31.4	79.5	810	34.4	na	unknown	2	M	26x20x18	1	0	0	0	0	1	3%	sparsely granulated corticotroph adenoma	99
C9	f	48	0	0	Cushig's disease	16.4	37	215	17.8	na	unknown	1	m	7x5x6	1	0	0	0	0	0	<3%	densly granulated corticotroph adenoma	95
C10	f	28	1	0	Cushig's disease	49.7	39	490	36.5	na	none	4	M	14x10x10	1	0	0	0	0	0	>5%	densly granulated corticotroph adenoma	99
C11	f	67	1	0	Cushig's disease	31	102	550	16.32	na	unknown	3	M	27 x 20 x 25	1	0	0	0	0	0	<3%	sparsely granulated corticotroph adenoma	95
C12	f	33	0	1	Cushig's disease	21.4	49	547	11.9	0.41	none	1	M	11x11x15	1	0	0	0	0	0	<3%	densly granulated corticotroph adenoma	90
C13	f	42	1	0	Cushig's disease	31.4	37.3	498	18	na	none	1	M	8x11x12	1	0	0	0	0	0	<1%	densly granulated corticotroph adenoma	99
C14	f	76	0	0	Cushig's disease	23.6	89	298	21.9	na	none	3	M	27x22x17	1	0	0	0	0	0	>3%	sparsely granulated corticotroph adenoma	99
C15	f	59	1	0	Cushig's disease	26.3	86.9	530	24.2	na	none	1	m	5x4x5	1	0	0	0	0	0	<1%	sparsely granulated corticotroph adenoma	100
C16	f	51	0	0	Cushig's disease	31.9	107	610	23.5	na	none	0	m	5x5x5	1	0	0	0	0	0	<1%	sparsely granulated corticotroph adenoma	97
C17	f	43	0	1	Cushig's disease	23.7	39.1	448	19	na	none	0	m	4x4x4	1	0	0	0	0	0	<1%	densly granulated corticotroph adenoma	99
C18	f	58	0	0	Cushig's disease	11.9	87	348	14.7	0.28	none	2	M	20x15x26	1	0	0	0	0	0	<1%	densly granulated corticotroph adenoma	99
C19	f	23	1	0	Cushig's disease	21.8	48.2	719	23.4	na	none	0	M	11x9x9	1	0	0	0	0	0	>10%	densly granulated corticotroph adenoma	99
C20	f	36	1	0	Cushig's disease	39.2	63.1	387	11.6	na	none	1	m	7x7x7	1	0	0	0	0	0	>3%	densly granulated corticotroph adenoma	99
C21	f	31	0	0	Cushig's disease	33.4	39.6	299	26.5	na	none	1	M	14x16x15	1	0	0	0	0	1	<1%	densly granulated corticotroph adenoma	99
C22	f	30	0	0	Cushig's disease	28.4	43.2	510	19.8	na	none	1	m	7x7x7	1	0	0	0	0	0	>3%	densly granulated corticotroph adenoma	99
C23	f	35	1	0	Cushig's disease	21	48.3	406	24.1	0.29	none	1	M	12x11x11	1	0	0	0	0	0	<3%	densly granulated corticotroph adenoma	97
C24	f	52	1	0	Cushig's disease	26.3	42.7	390	19.3	na	none	1	m	6x6x7	1	0	0	0	0	1	<3%	densly granulated corticotroph adenoma	98
C25	f	34	1	0	Cushig's disease	21.6	39.3	276	23.8	na	none	0	m	4x4x4	1	0	0	0	0	1	<1%	densly granulated corticotroph adenoma	80
C26	m	71	0	0	Cushig's disease	36.2	129	298	21.1	na	none	0	M	21x20x16	1	0	0	0	0	0	<1%	sparsely granulated corticotroph adenoma	100
C27	f	67	1	0	Cushig's disease	21.9	43.1	319	13.9	na	none	3	m	3x4x9	1	0	0	0	0	0	<1%	densly granulated corticotroph adenoma	100
C28	f	41	0	0	Cushig's disease	27.1	93.6	284	15.3	na	unknown	1	m	7x6x5	1	0	0	0	0	0	<1%	densly granulated corticotroph adenoma	98
S1	m	57	0	0	Silent	11.4	74.9	117	0.8	0,01; 0,01	none	3	M	24x33x28	1	0	0	0	0	0	<3%	sparsely granulated corticotroph adenoma	95
S2	m	54	0	0	Silent	11.3	57.3	27.3	0.8	0,01;0,009	none	2	M	26x19x21	1	0	0	0	0	0	<3%	sparsely granulated corticotroph adenoma	100
S3	m	53	0	0	Silent	21.4	51.3	108	1.1	0,01;0,01	none	4	M	28x24x19	1	0	0	0	0	0	<1%	densly granulated corticotroph adenoma	99
S4	m	55	0	0	Silent	27.1	29.1	39.8	0.3	na	none	1	M	12x12x15	1	0	0	0	0	0	<1%	sparsely granulated corticotroph adenoma	90
S5	m	77	0	0	Silent	19.7	62.3	47.2	1.7	0,03;0,03	none	1	M	21x19x13	1	0	0	0	0	0	<3%	densly granulated corticotroph adenoma	95
S6	f	54	1	0	Silent	9.1	47.5	124	1.9	na	none	2	M	25x28x21	1	0	0	0	0	0	<3%	densly granulated corticotroph adenoma	99
S7	m	45	0	0	Silent	12.1	32.1	110	0.9	na	none	1	M	34x25x20	1	0	0	0	0	0	3%	sparsely granulated corticotroph adenoma	99
S8	f	64	0	0	Silent	19.2	46.7	29.8	0.9	na	none	2	M	25x18x15	1	0	0	0	0	1	>5%	densly granulated corticotroph adenoma	100
S9	m	44	0	0	Silent	21.3	57.9	126	1.1	0,03;0,02	none	1	M	20x25x32	1	0	0	0	0	0	<3%	densly granulated corticotroph adenoma	90
S10	f	43	0	0	Silent	7.8	39.7	13.7	0.4	0,02;0,05	none	1	M	21x14x21	1	0	0	0	0	0	>3%	sparsely granulated corticotroph adenoma	90
S11	f	34	0	0	Silent	19.7	63.9	63.84	0.8	na	none	0	M	21x22x15	1	0	0	0	0	0	<3%	densly granulated corticotroph adenoma	99
S12	f	42	0	0	Silent	12.9	36.7	111.7	1.9	0,03;0,02	none	2	M	17x25x31	1	0	0	0	0	0	<3%	sparsely granulated corticotroph adenoma	98
S13	f	23	1	0	Silent	19.9	42.1	139	1.3	0,06;0,03	none	0	M	19x15x13	1	0	0	0	0	0	>10%	sparsely granulated corticotroph adenoma	99
S14	f	61	0	0	Silent	20.1	61.2	97.4	0.4	0,09;0,02	none	1	M	15x12x14	1	0	0	0	0	1	<3%	sparsely granulated corticotroph adenoma	99
S15	m	54	0	0	Silent	17.1	51.3	59.8	0.4	na	none	2	M	21x18x17	1	0	0	0	0	0	<1%	densly granulated corticotroph adenoma	99
S16	f	69	0	0	Silent	29.7	41.3	37.91	0.9	na	none	4	M	19x25x28	1	0	0	0	0	0	<3%	sparsely granulated corticotroph adenoma	98
S17	f	60	0	0	Silent	6.8	14.7	123	1.4	na	none	3	M	24 x18x20	1	0	0	0	0	0	3%	sparsely granulated corticotroph adenoma	98
S18	m	61	0	0	Silent	10.9	21.8	68.8	4.9	na	none	1	M	20x14x15	1	0	0	0	0	0	<3%	sparsely granulated corticotroph adenoma	99
S19	f	29	1	0	Silent	50.8	61.3	94.76	0.4	0,02;0,04	none	1	M	17x12x18	1	0	0	0	0	0	<1%	densly granulated corticotroph adenoma	100
S20	f	77	1	0	Silent	13.6	47.9	124	1.4	na	none	1	M	12x15x10	1	0	0	0	0	0	<1%	sparsely granulated corticotroph adenoma	98

Table S2: Gene set over-representation analysis (GSOA); Top 20 pathways enriched for the genes that are targets of differentially expressed miRNAS

GO molecular function

	GO molecular function term	fold Enrich	p-value	adjusted p-value
1	steroid hormone receptor activity (GO:0003707)	7.86	1.87E-04	1.75E-02
2	nuclear receptor activity (GO:0004879)	5.2	9.24E-07	1.42E-04
3	ligand-activated transcription factor activity (GO:0098531)	5.2	9.24E-07	1.34E-04
4	regulatory RNA binding (GO:0061980)	5.06	2.95E-04	2.66E-02
5	transcription coregulator binding (GO:0001221)	3.6	1.31E-04	1.27E-02
6	phosphatase binding (GO:0019902)	3.09	7.99E-05	8.03E-03
7	DNA-binding transcription repressor activity, RNA polymerase II-specific (GO:000122	3.09	1.98E-05	2.25E-03
8	DNA-binding transcription repressor activity (GO:0001217)	2.98	3.11E-05	3.39E-03
9	DNA-binding transcription activator activity (GO:0001216)	2.5	6.56E-07	1.07E-04
10	DNA-binding transcription activator activity, RNA polymerase II-specific (GO:0001228	2.47	1.12E-06	1.55E-04
11	RNA polymerase II cis-regulatory region sequence-specific DNA binding (GO:0000978	2.27	2.37E-09	3.10E-06
12	cis-regulatory region sequence-specific DNA binding (GO:0000987)	2.27	1.72E-09	4.50E-06
13	RNA polymerase II-specific DNA-binding transcription factor binding (GO:0061629)	2.25	3.92E-04	3.41E-02
14	DNA-binding transcription factor activity (GO:0003700)	2.22	4.25E-09	2.78E-06
15	DNA-binding transcription factor activity, RNA polymerase II-specific (GO:0000981)	2.2	1.03E-08	4.48E-06
16	RNA polymerase II transcription regulatory region sequence-specific DNA binding (G	2.2	5.63E-09	2.95E-06
17	transcription factor binding (GO:0008134)	2.13	2.50E-06	3.27E-04
18	transcription cis-regulatory region binding (GO:0000976)	2.09	1.27E-08	4.74E-06
19	transcription regulatory region nucleic acid binding (GO:0001067)	2.08	1.34E-08	4.38E-06
20	sequence-specific double-stranded DNA binding (GO:1990837)	2.01	4.28E-08	1.02E-05

GO biological process

	GO biological process term	fold Enrich	p-value	adjusted p-value
1	negative regulation of stem cell differentiation (GO:2000737)	8.24	4.64E-04	2.40E-02
2	neuronal stem cell population maintenance (GO:0097150)	7.21	2.77E-04	1.67E-02
3	response to muscle stretch (GO:0035994)	5.77	7.72E-04	3.54E-02
4	maintenance of cell number (GO:0098727)	4.72	6.49E-08	1.49E-05
5	steroid hormone mediated signaling pathway (GO:0043401)	4.63	5.65E-05	4.45E-03

6	stem cell population maintenance (GO:0019827)	4.52	4.96E-07	7.41E-05
7	hormone-mediated signaling pathway (GO:0009755)	4.47	2.29E-06	2.71E-04
8	somatic stem cell population maintenance (GO:0035019)	4.45	6.28E-04	3.01E-02
9	negative regulation of fat cell differentiation (GO:0045599)	4.28	7.92E-04	3.59E-02
10	neural crest cell development (GO:0014032)	4.25	2.18E-04	1.37E-02
11	lung morphogenesis (GO:0060425)	4.12	9.88E-04	4.27E-02
12	associative learning (GO:0008306)	4.12	5.17E-04	2.56E-02
13	neural crest cell differentiation (GO:0014033)	4.01	1.76E-04	1.19E-02
14	mesenchymal cell development (GO:0014031)	4	3.36E-04	1.89E-02
15	stem cell development (GO:0048864)	3.88	4.13E-04	2.25E-02
16	regulation of Rho protein signal transduction (GO:0035023)	3.86	7.86E-04	3.57E-02
17	intracellular receptor signaling pathway (GO:0030522)	3.76	1.46E-06	1.86E-04
18	post-embryonic development (GO:0009791)	3.74	9.59E-04	4.18E-02
19	response to estrogen (GO:0043627)	3.74	9.59E-04	4.17E-02
20	positive regulation of pri-miRNA transcription by RNA polymerase II (GO:1902895)	3.67	6.12E-04	2.95E-02

Table S3: Sequences of qRT-PCR primers

primers for human cDNA ampliification		
POMC	AGGACCTCACCACGGAAAG	GGGAACATGGGAGTCTCGG
NR3C1	TGCTCCTTCTGCGTTCACAA	CCATCAGTGAATATCAACTCTGGC
NR3C2	GGGGATGAGGCTTCAGGATG	AGTTGTGTTGCCCTTCCACT
GAPDH	GAAGATGGTGATGGGATTTC	GAGGTGAAGGTCGGAGTC

primers for mouse cDNA ampliification		
NR3C1	TGGAGAGGACAACCTGACTTCC	ACGGAGGAGAACTCACATCTGG
NR3C2	AACCACACGGTGACCTGTCATC	AGTGACACCCAGAAGCCTCATC
GAPDH	CATCACTGCCACCCAGAAGACTG	ATGCCAGTGAGCTTCCCGTTCAG
